# Antimicrobial Photodynamic Inactivation Mediated by Rose Bengal and Erythrosine Is Effective in the Control of Food-Related Bacteria in Planktonic and Biofilm States

**DOI:** 10.3390/molecules23092288

**Published:** 2018-09-07

**Authors:** Alex Fiori Silva, Anabela Borges, Camila Fabiano Freitas, Noboru Hioka, Jane Martha Graton Mikcha, Manuel Simões

**Affiliations:** 1 Postgraduate Program of Health Sciences, State University of Maringá, Av. Colombo, 5790, Maringá 87020-900, Paraná, Brazil; alex_skiba@hotmail.com (A.F.S.); jmgmikcha@uem.br (J.M.G.M.); 2 LEPABE, Department of Chemical Engineering, Faculty of Engineering, University of Porto, Rua Dr. Roberto Frias, s/n, 4200-465 Porto, Portugal; apborges@utad.pt; 3 Department of Chemistry, State University of Maringa, Av. Colombo, 5790, Maringá 87020-900, Paraná, Brazil; camila.freitas1989@hotmail.com (C.F.F.); nhioka@uem.br (N.H.)

**Keywords:** biofilms, erythrosine, food-related bacteria, light emitting diode, photodynamic inactivation, rose bengal

## Abstract

The thermal and chemical-based methods applied for microbial control in the food industry are not always environmentally friendly and may change the nutritional and organoleptic characteristics of the final products. Moreover, the efficacy of sanitizing agents may be reduced when microbial cells are enclosed in biofilms. The objective of this study was to investigate the effect of photodynamic inactivation, using two xanthene dyes (rose bengal and erythrosine) as photosensitizing agents and green LED as a light source, against *Staphylococcus aureus*, *Listeria innocua*, *Enterococcus hirae* and *Escherichia coli* in both planktonic and biofilm states. Both photosensitizing agents were able to control planktonic cells of all bacteria tested. The treatments altered the physicochemical properties of cells surface and also induced potassium leakage, indicating damage of cell membranes. Although higher concentrations of the photosensitizing agents (ranging from 0.01 to 50.0 μmol/L) were needed to be applied, the culturability of biofilm cells was reduced to undetectable levels. This finding was confirmed by the live/dead staining, where propidium iodide-labeled bacteria numbers reached up to 100%. The overall results demonstrated that photoinactivation by rose bengal and erythrosine may be a powerful candidate for the control of planktonic cells and biofilms in the food sector.

## 1. Introduction

Outbreaks of foodborne diseases are an international public health problem and an important cause of reduced economic growth [1]. According to the World Health Organization it is estimated that more than 600 millions of people get sick as result of unsafe food consumption [2]. Only in the United States 48 millions of foodborne illness cases are reported every year, with 3000 deaths [3].

One of the most common source of food contamination is related to the occurrence of biofilms on food-related surfaces [4]. Biofilms are characterized as an assemblage of surface-associated microorganisms, typically bacteria, enclosed in a self-produced polymeric matrix [5]. Besides food contamination, the presence of microbial biofilms in food processing environments induces other problems, including damage of metal surface pipelines (e.g., corrosion), reduction of the heat transfer in heat exchange equipment, reduction of the permeability of filtrations systems and even mechanical blockage [6]. In addition, sessile cells are more tolerant to the sanitizing agents used for microbial control [7,8].

Although the current thermal and chemical-based procedures have been extensively applied in the food industry for controlling microorganisms, several limitations are recognized. For instance, thermal procedures need an external heat source [9] and may induce changes in the sensorial and nutritional properties of foodstuffs [10]. Regarding the chemical sanitizers, the use of chlorine-based sanitization can induce corrosion on equipment surfaces and, when in the presence of organic substances, the generation of organochlorinated carcinogenic and mutagenic by-products can occur [11]. Therefore, the development of new strategies for controlling microorganisms in the food sector is a research need. There is increasing evidence that antimicrobial photodynamic inactivation (aPDI) can be a powerful tool for this purpose [12,13,14,15]. This technique is based on the excitation of a non-toxic dye—called photosensitizer (PS)—by irradiation with visible light of a suitable wavelength, in the presence of oxygen. The excitation of the PS triggers the generation of reactive oxygen species (ROS) that are able to react with cellular components (nucleic acids, proteins, lipids) and lead to microbial inactivation [16]. Interestingly, this method is not related to the induction of antimicrobial resistance, is eco-friendly and relatively cheap to implement [17,18]. These aspects reinforce the attractiveness of aPDI for the food sector.

In view of the limited knowledge on the effectiveness of aPDI focused on the food area, especially regarding biofilm control, the necessity of expanding knowledge on this field is unquestionable. In this study, four food-related bacteria, *Staphylococcus aureus*, *Listeria innocua*, *Enterococcus hirae* and *Escherichia coli*, were used as a model to evaluate the antimicrobial effectiveness of rose bengal (RB) and erythrosine (ERY), two dyes approved for food usage, upon illumination with a light emitting diode (LED) source. The antibiofilm activity of both dyes was also studied. To the best of our knowledge, this is the first study evaluating the antibiofilm effectiveness of RB and ERY using green LED light source against *L. innocua* and *E. hirae*.

## 2. Results

### 2.1. Light Doses

**Figure 1:**
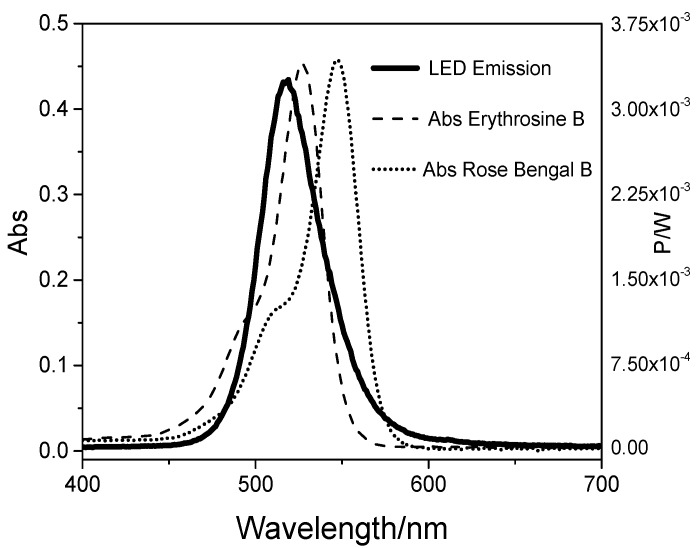
Light-emitting diode emitted potency (P_LED_ emitted) and absorbed potency (P_Abs_) by ERY and RB.

The emission spectrum of the LED light source and the absorption spectra of RB and ERY are depicted in Figure 1. The data demonstrate an adequate match between the LED and the spectra of both PSs.

**Figure 2:**
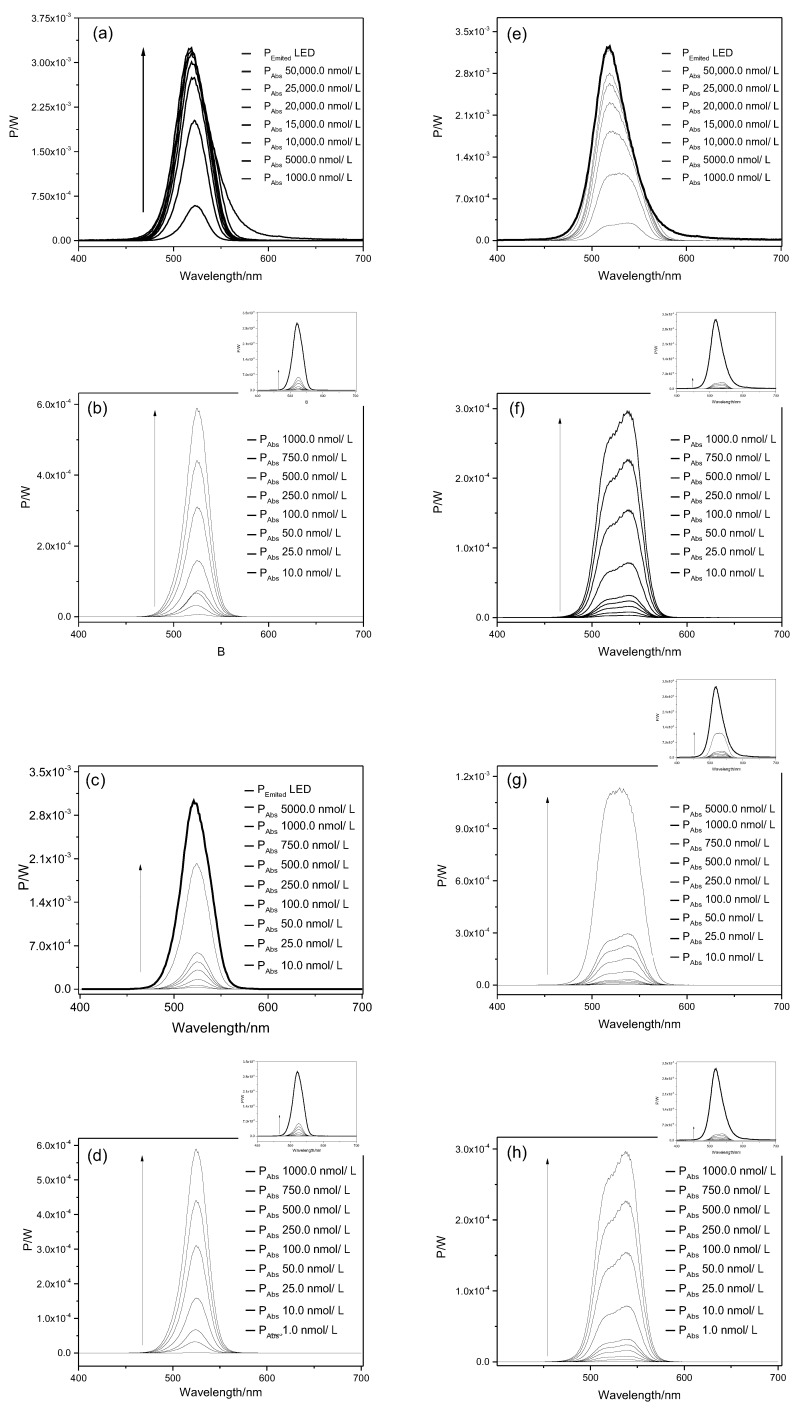
Spectra of light emitted by LED and power absorbed by ERY and RB for planktonic cells of *E. coli* (**a**,**e**), *S. aureus* (**b**,**f**), *E. hirae* (**c**,**g**) and *L. innocua* (**d**,**h**). The arrow indicates increase in photosensitizer concentration. The small chart displayed on the top-right side of figures (**b**,**d**,**f**,**g**,**h**) corresponds to its scaled-down graph.

**Figure 3:**
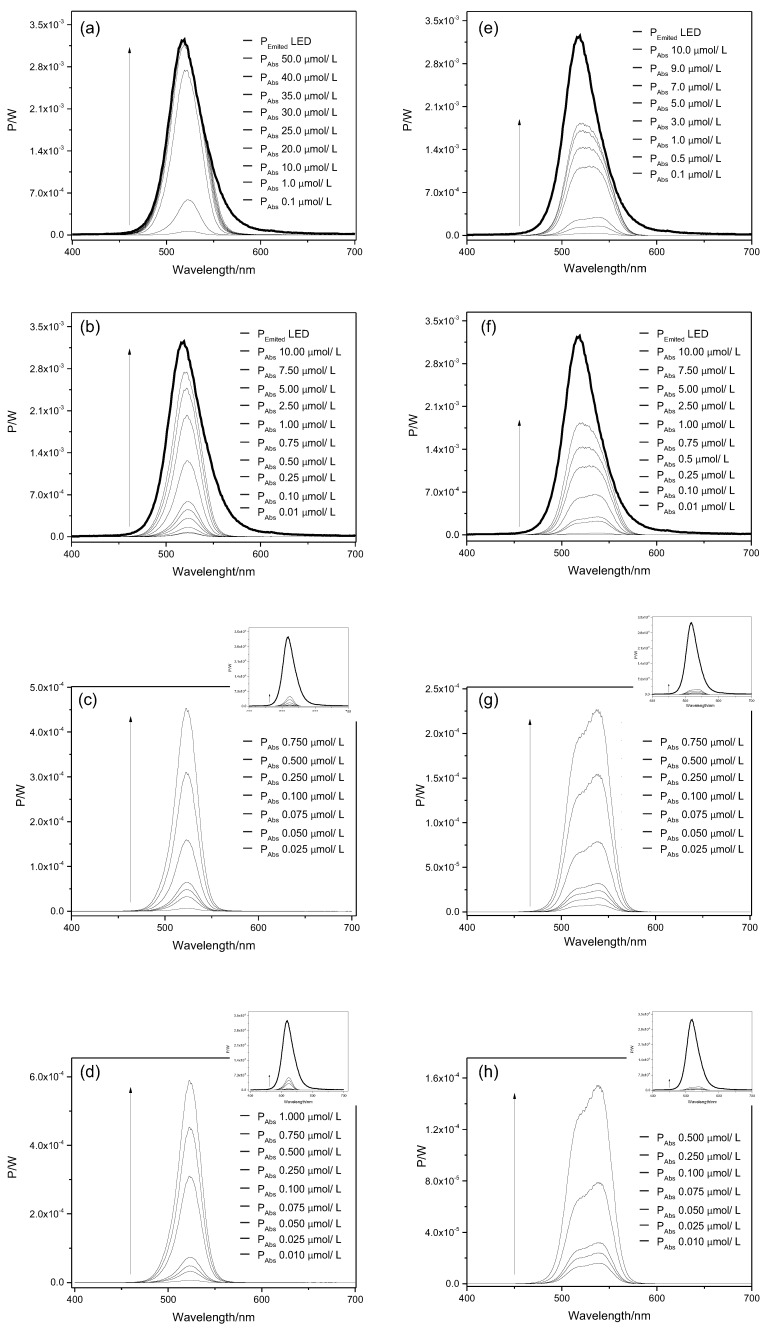
Spectra of light emitted by LED and power absorbed by ERY and RB for biofilm cells of *E. coli* (**a**,**e**), *S. aureus* (**b**,**f**), *E. hirae* (**c**,**g**) and *L. innocua* (**d**,**h**). The arrow indicates increase in photosensitizer concentration. The small chart displayed on the top-right side of figures (**c**,**d**,**g**,**h**) corresponds to its scaled-down graph.

The values of light dose to apply against both planktonic cells and biofilms were obtained from Equations (1) and (2) and the results are shown in Table 1. The irradiation parameters were also adjusted for polychromatic light according to the numbers of photons absorbed as described previously [19,20] (Supplementary Table S1). It can be noticed that the light dose increases proportionally to the PS concentration. For instance, the light dose for ERY at 10 and 50,000 nmol/L was found to be 0.22 and 162.07 J/cm^2^, respectively. For RB at the same concentrations the light doses were 0.17 and 166.44 J/cm^2^, respectively. Regarding light doses for biofilm treatment, it was observed that the values obtained were higher than those found for the planktonic cells. For instance, the light dose for ERY at 10 nmol/L is 0.22 J/cm^2^ for planktonic cells (10 min of light exposure), while light doses of 0.65 J/cm^2^ are found for the same concentration of ERY for biofilm experiments. The spectra of light emitted by LED (P_LED_ emitted) and power absorbed by PSs (P_Abs_) for different concentrations is given in Figure 2 and Figure 3.

### 2.2. Effect of RB and ERY Photosensitizers on Planktonic Cells

**Figure 4:**
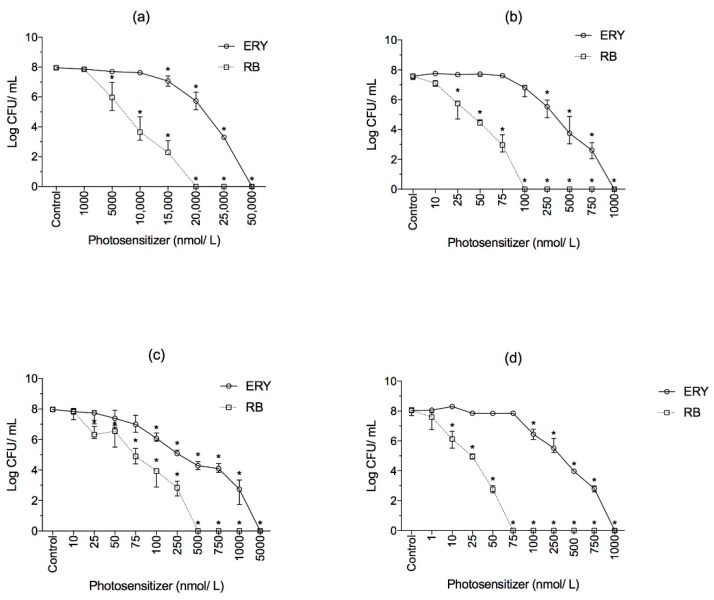
Survival of (**a**) *E. coli*, (**b**) *S. aureus*, (**c**) *E. hirae* and (**d**) *L. innocua* planktonic cells subjected to aPDI using different concentrations of ERY and RB. Cells were incubated for 10 min in the dark and then illuminated with green LED light source for 10 min. The control group represents the cells incubated only with PBS. Values are shown as medians, including 25% and 75% quantiles of at least three independent experiments. * *p* < 0.05 compared to the control.

No antimicrobial effects were observed when planktonic bacteria were kept either in the dark (20 min; dark control) with PS or exposed to LED light for 20 min (without PS; light control) (Supplementary Figure S1). However, the combination of PS and illumination with green LED significantly reduced the culturability of all bacteria tested (*p* < 0.05; Figure 4). It is clear that the antimicrobial effectiveness of photodynamic inactivation (PDI) is strongly dependent on the PS concentration and on the microorganism. Among the bacteria tested, *E. coli* was the less susceptible to aPDI since higher light doses (RB—139.46 J/cm^2^; ERY—162.07 J/cm^2^) were needed to inactive this bacterium. Regarding Gram-positive bacteria, the susceptibility to aPDI decreased in the following order: *L. innocua* > *S. aureus* > *E. hirae*.

#### 2.2.1. Physicochemical Characterization of the Bacterial Surfaces

The hydrophobicity and the surface tension parameters of photoinactivated-bacteria were investigated based on contact angle measurements. As shown in Table 2, all bacteria presented hydrophilic character (ΔG_iWi_ > 0) and were predominantly electron donors and less prone to accept electrons. The aPDI mediated by ERY and RB decreased *S. aureus* hydrophobicity from 29.57 mJ/m^2^ to 21.76 and 22.44 mJ/m^2^ (*p* < 0.05), respectively. The ability of *S. aureus* to donates electron was also reduced by the treatment with both dyes. *E. coli* apolar component increased while the polar component decreased from RB and ERY aPDI. The surface parameters of *E. hirae* and *L. innocua* were less affected by aPDI (*p* > 0.05).

#### 2.2.2. Intracellular Potassium Leakage

Table 3 shows the effect of aPDI mediated by ERY and RB on K^+^ leakage by *E. coli*, *S. aureus*, *E. hirae* and *L. innocua*. No K^+^ leakage was observed when cells were submitted only to irradiation or only to PS. On the other hand, photoinactivation induced K^+^ leakage for all bacteria tested (*p* < 0.05; Table 3). The K^+^ leakage from Gram-positive bacteria was higher compared to the *E. coli*. In fact, *E. coli* presented the smallest content release from both ERY and RB treatments (0.64 and 0.20 mg/L, respectively). The highest K^+^ release was observed for *S. aureus* (~1.22 mg/L) and for both PSs.

### 2.3. Effect of aPDI on Biofilms

**Figure 5:**
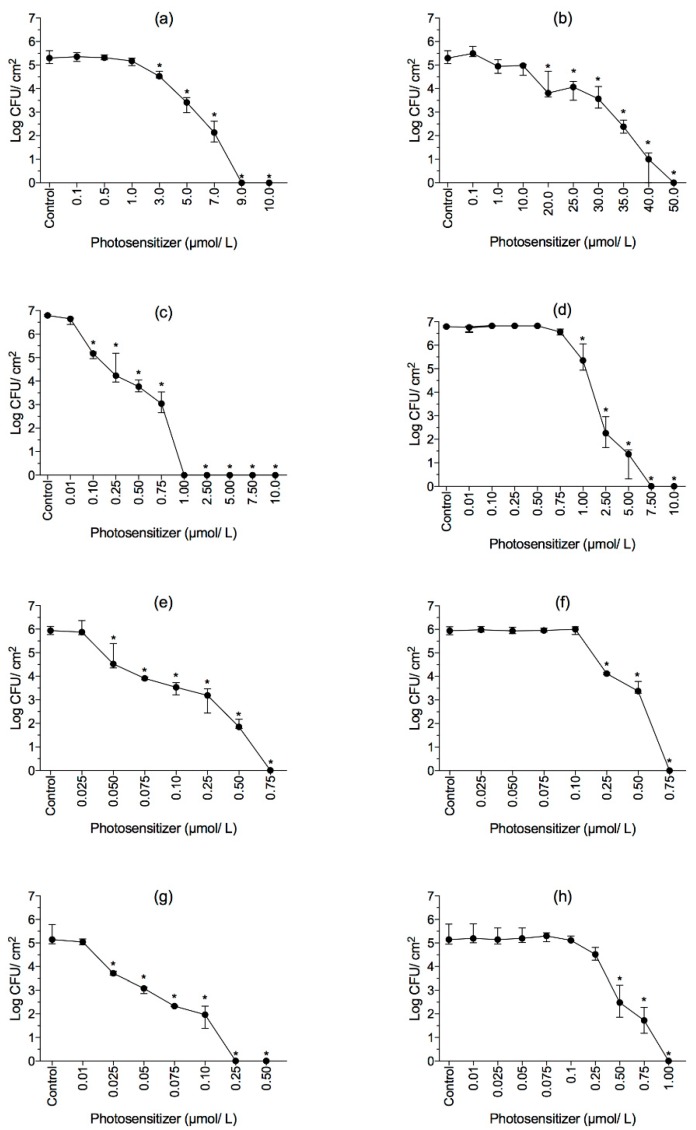
The survival of biofilms cells formed by (**a**,**b**) *E. coli*, (**c**,**d**) *S. aureus*, (**e**,**f**) *E. hirae* and (**g**,**h**) *L. innocua* subjected to aPDI using RB (left) and ERY (right). Biofilms were incubated for 30 min in the dark and illuminated with green LED light source for 30 min. The control group represents the cells incubated only with PBS. Values are shown as medians, including 25% and 75% quantiles of at least three independent experiments. * *p* < 0.05 compared to the control.

No antibiofilm effects were observed from exposure the dyes alone or to the light alone (Supplementary Figure S2). After dark incubation with increasing PS concentrations following illumination, the biofilm viability decreased significantly (Figure 5 and Table 1). The minimal biofilm eradication concentrations (MBEC) of RB were 0.25, 0.75, and 1.0 μmol/L (light doses: 12.99, 38.00 and 49.59 J/cm^2^) for *L. innocua*, *E. hirae* and *S. aureus*, respectively (*p* < 0.05; Figure 5). Higher concentrations of ERY were needed to reach the same results for *L. innocua* (1.0 μmol/L), *E. hirae* (0.75 μmol/L) and *S. aureus* (7.5 μmol/L) biofilms (Figure 5; *p* < 0.05). *E. coli* produced the most photoresistant biofilm, where light doses delivery of 486.40 J/cm^2^ (50 μmol/L) and 291.08 J/cm^2^ (9 μmol/L) for ERY and RB, respectively, were needed to reduce biofilm cells to undetectable levels. In general, RB was more effective in biofilm control than ERY (*p* < 0.05).

### 2.4. Effect of aPDI on Membrane Integrity of Biofilms

The membrane integrity of biofilm subjected to aPDI was also assessed by fluorescence microscopy using Live/Dead assay (Table 4). The light doses used for biofilm treatments are presented in Table 1. The lowest concentration (0.01, 0.01, 0.025 and 0.10 μmol/L, for *L. innocua*, *S. aureus*, *E. hirae* and *E. coli*, respectively; Figure 5) of both PS employed caused negligible or no effect on membrane integrity of biofilm cells (data not shown). On the other hand, when ERY was used at MBEC, the number of PI-stained cells of *S. aureus*, *E. hirae* and *L. innocua* reached up to 100% (*p* < 0.05), while 98.19% of *E. coli* population were stained with PI (*p* < 0.05; Table 4). The aPDI mediated by RB at MBEC caused 100% of PI uptake by *S. aureus* and *E. hirae* (*p* < 0.05) and ~99% for both *E. coli* and *L. innocua* cells.

## 3. Discussion

PDI is increasingly being considered an emergent strategy for controlling pathogens in the food sector due to its safe, fast and inexpensive status [17]. Moreover, the application of natural molecules with photosensitizing properties already approved to be used in the food industry, make this strategy even more attractive. The present study focused on the evaluation of aPDI on the control of food-related bacteria in both planktonic and sessile states. For that two nontoxic dyes, ERY and RB, were used with green LED light irradiation.

The range of light absorption of RB is 480–580 nm while ERY absorbs light in the range of 450–550 nm. The peak absorption is 557 and 532 nm for RB and ERY, respectively. It is noticeable that the spectra emission from the LED light system used overlapped the RB and ERY absorption spectrum. Such feature allows the effective activation of the PSs, reducing the necessity to reduce the distance between the light device and the specimen, and the use of high light doses [21]. Moreover, the green light system applied revealed to be adequate for RB and ERY activation.

RB and ERY exhibit different spectrum profiles, which can change the absorption of light intensity of each PS. Therefore, Equations (1) and (2) were applied to obtain the appropriate light dose values, according to the light fluency absorbed by the PS at different concentrations. It was demonstrated that the PS absorption spectrum increases proportionally with its concentration and differs between the two dyes. This results is in accordance with previous studies [12,22] and highlight the importance of taking into account the type of PS in light dose calculations, which are frequently neglected in most of the studies evaluating aPDI effectiveness [23,24,25]. A reasonable explation for this finding is provided by the equations demonstrated in the Supplementary Material, where the light dose absorbed (D_Abs_) by each photosensitizer is obtained by relating the illumination time (*t*), the irradiated area (A) and the absorbed power (P_Abs_) Equation (S11). As shown in Equations (S7) and (S8), the P_Abs_ is obtained by the relationship between the emitted power by the light source and the fraction absorbed (X_Abs_) by the photosensitizer Equations (S7) and (S8). The X_Abs_ corresponds to the difference of the light fraction that crossed the sample, and is obtained through Equation (S5): $X_{\mathrm{Abs}}=1-10^{-\mathrm{Abs}}$. Once the D_Abs_ by the photosensitizer is dependent on its absorption and, therefore, on its concentration, it is expected that the ligh dose values increase with the increase of PS concentrations.

In vitro aPDI of planktonic cells revealed no culturable *S. aureus* when 1000 nmol/L (20.52 J/cm^2^) ERY was applied. This result is similar to other studies evaluating the photoinactivation of *S. aureus* using ERY as PS [22,26]. *E. hirae* was also found to be completely eliminated upon PDI with 500 nmol/L RB and 10 min of illumination (8.55 J/cm^2^). On the other hand, in a study conducted by Ergaieg and Seux [27], 0.73 μmol/L RB followed by ~30 min of artificial sunlight was necessary to reduced *E. hirae* cells to undetectable counts. The longer light exposure needed to achieve complete inactivation of *E. hirae* by Ergaieg and Seux [27] may be attributed to the relative absence of spectral overlap of the respective light emission spectra and the absorption spectra of PS.

In this study, *L. innocua* was found to be the most susceptible bacterium to PDI among the Gram-positive ones. Low PS concentrations were able to reduce *L. innocua* to undetectable levels compared to the other bacteria tested. Previous studies also demonstrated that the photokilling process is strain-dependent [28], being mostly affected by the bacterial permeability to the PS [29].

Comparing the photoinactivation between the bacteria tested, it is clear that *E. coli* was more tolerant to photosensitization than the Gram-positive ones. Our results are supported by other studies [12,15,22] where it was also observed that higher PS concentrations or longer irradiation times were needed to be applied against Gram-negative bacteria. Such tolerance to aPDI is attributed to the differences in cell envelope structure between the two types of bacteria. The outer membrane of Gram-negative bacteria is supposed to reduce the degree of permeability to some PS [30,31] and plays an important role in aPDI resistance.

The changes induced by PDI on physicochemical properties of the bacterial surface were assessed by contact angles measurements using sessile drop method. According to van Oss [32], a given surface is considered to be hydrophobic if the ΔG_iWi_ is < 0, and hydrophilic when ΔG_iWi_ is > 0. Thus, all bacteria evaluated presented hydrophilic surfaces properties. Generally, the bacteria tested also presented high values (~52 mJ/m^2^) for the electron donor parameter (γ^−^). This may be attributed to the neutralization of γ^+^ induced by oxygen molecules in the surface of the bacteria [32]. Moreover, it was observed that ΔG_iWi_ of the selected bacteria was affected by PDI. For instance, both RB and ERY increased the hydrophobicity of *E. coli* cells, while all other Gram-positive bacteria presented lower ΔG_iWi_ values after photoinactivation. This phenomenon is probably related to changes of the hydrophobic attribute of lipopolysaccharides (LPS) present on outer and cytoplasmic membranes, which can induce the death of bacterial cells [33]. The apolar, polar and electron acceptor components of *E. coli* and *S. aureus* were altered in a great manner by aPDI. Taking into account that biomolecules (e.g., proteins and lipids) are targeting agents of ROS generated during aPDI [34], it may be considered that the modifications of the physicochemical properties of bacterial surfaces observed after aPDI is able to disturb the amphiphilic characteristic of the lipid bilayer and cause bacterial growth inhibition or death [33]. It was also proposed that after an initial binding, the PSs accumulate on the bacterial membrane [35], changing its physicochemical characteristics and inducing cell death [36]. Regarding *E. hirae* and *L. innocua*, it was observed that its surface tensions parameters were affected in a lower extent. This finding suggests that although changes on physiochemical properties of these bacteria were observed, it may not be the only mechanism behind the antimicrobial effectiveness of aPDI.

To further assess the cytoplasmic membrane damage after PDI, the release of K^+^ was measured. This is the main ion within cells and is essential for several functions [37]. It has been described that K^+^ leakage is a useful indicator for monitoring integrity of microorganisms [38]. In this study, K^+^ leakage was observed for all bacteria tested when PS was combined with irradiation. The results obtained corroborate the cell surface changes observed from contact angles measurements and are in agreement with another studies [39] where K^+^ leakage was observed after aPDI. Xanthene dyes are amphiphilic molecules and according to the partition coefficients they present lipophilic tendency [40]. This proposes the partition of the compounds into the cell membrane, leading to the K^+^ leakage as a consequence of membrane instability and the breakdown of the permeability barrier. This finding reinforces that membrane damaged may play an important role aPDI mode of action [41,42].

Regarding aPDI on biofilms, it was observed that sessile cells were successfully inactivated in a light dose-depend manner. It is clear that *E. coli* presented the highest tolerance to aPDI. For instance, the treatment with 50 μmol/L (486.40 J/cm^2^) ERY completely inactivated *E. coli* biofilms, while *S. aureus*, *E. hirae* and *L. innocua* biofilms were reduced to undetectable levels using lower light doses of 295.83, 47.31 and 61.56 J/cm^2^, respectively. Similar behaviors were also observed to aPDI mediated by RB, where light doses of 291.08, 49.5, 38.0 and 12.99 J/cm^2^ completely inactivated *E. coli*, *S. aureus*, *E. hirae* and *L. innocua* biofilms, respectively. These results are consistent with other studies [43,44], where biofilms formed by *S. aureus* were more susceptible to photoinactivation mediated by methylene blue and toluidine blue than *E. coli* biofilms. In a previous study [45], ~20% of *E. coli* O157:H7 biofilms cells remained metabolically active after treatment with gallic acid combined with UV-A light. In a study conducted by Ronqui et al. [43] a higher concentration of methylene blue (200 μg/mL) was needed to decrease 4 log CFU/mL of *E. coli* biofilms. Although the aforementioned studies [43,45] demonstrated the decrease of cell counts/metabolic activity of *E. coli* biofilms, a complete inactivation was not achieved when gallic acid and methylene blue were used. Such findings in conjugation with the results from the present study reinforce that aPDI mediated by RB and ERY in combination with green LED light may be an effective tool for controlling bacterial biofilms.

*S. aureus* biofilms were reduced to undetectable levels when 1 μmol/L (49.59 J/cm^2^) RB or 7.5 μmol/L (295.83 J/cm^2^) ERY were applied. This finding is in agreement with Ke et al. [46], where no cultivable cells were recovered after PDI mediated by 0.05 mmol/L of ERY activated by a LED light device. Di Poto et al. [47] evaluated the effectiveness of the tetra-substituted *N*-methyl-pyridyl-porphine activated by a white light against biofilms formed by three distinct strains of *S. aureus*. In that study, cell viability was decreased by ~2 log with 10 μM PS and the higher light dose tested (200 J/cm^2^). In disagreement with the results found in the current study, Di Poto et al. [47] observed viable cells after aPDI, a result apparently related to the short time of dark incubation (15 min), which did not allow the appropriate PS penetration throughout the biofilm.

*L. innocua* biofilms were successfully photoinactivated by 0.25 μmol/L (12.99 J/cm^2^) RB and 1 μmol/L (61.56 J/cm^2^) ERY. Luksiene and Paskeviciute [48] found that *Listeria monocytogenes* biofilms on a food-related surface, polyolefine, were completely eliminated by 150 μmol/L Na-chlorophyllin followed by 5 min irradiation (λ = 405 nm). More recently, Bonifácio et al. [49] observed *L. monocytogenes* biofilms reductions from 1.3 × 10^7^ CFU/mL to 1.7 × 10^2^ after PDI with curcumin at 3.7 mg/L and 30 min of light exposure (λ = 400–500 nm). Notably, comparing the present results with the above-mentioned studies, higher PS concentrations were needed to cause inactivation of *Listeria* spp. biofilms, highlighting the high efficiency of ERY and RB activated by green LED light.

In the present study, biofilms formed by *E. hirae* were completely eliminated after treatments with ERY or RB at 0.75 μmol/L. Although some studies evaluating the PDI of *Enterococcus* spp. biofilms are available, according to our knowledge, this is the first report covering the antibiofilm properties of LED-light-activated RB and ERY against sessile cells of *E. hirae.* In a study carried out by Shrestha et al. [50], it was demonstrated that *Enterococcus faecalis* biofilms were reduced by 3 log CFU/mL when 10 μmol/L RB was irradiated with an optical fiber bundle. In another study [51] the combination of 100 μmol/L RB and 35 J/cm^2^ irradiation led to ~5 log CFU/mL reduction of *E. faecalis* biofilms. Unlike the results of previous reports [50,51], in the present study no culturable *E. hirae* were found after PDI with RB and ERY at low concentrations/light dose. Such difference may be explained by different susceptibilities of different *Enterococcus* strains and the differences in the experimental conditions used.

Live/dead staining revealed that biofilms subjected to aPDI presented cells mostly PI stained. Such results propose that membrane permeabilization may be an important target of aPDI [52]. Almost all bacteria tested were 100% of PI stained after photoinactivation, with exception of *E. coli* for RB and ERY treatments and *L. innocua* for RB, where ~98% of the cells PI stained. The 1% remaining green-cells were not recovered in CFU counts. A possible explanation is that a sub-lethal viable but non-culturable (VBNC) state was induced by PDI, in which cells cannot be detected by conventional plating methods [53].

The overall results show that biofilm cells were less susceptible to PDI compared to the planktonic ones. Several mechanisms have been purposed to explain the photoresistance of biofilms, including the neutralization or reduced diffusion of singlet oxygen through the biofilm matrix [54]; the limited penetration of light [55] and PS which leads to its accumulation on the biofilm upper layers [28]. Hence, higher PS concentrations and light doses were necessary to cause complete inactivation of sessile cells compared to the planktonic ones.

Among the two PS tested, RB was found to be more effective than ERY. This result is in agreement with other studies evaluating the antimicrobial effectiveness of aPDI mediated by xanthene dyes (e.g., RB, eosin Y, ERY) [26,56,57,58]. This finding may be attributed to the capacity of RB in producing singlet oxygen with a quantum yield of nearly 100% due to the number of iodide substituents in the xanthene ring under irradiation [59,60].

## 4. Materials and Methods

### 4.1. Bacterial Strains and Culture Conditions 

The Gram-negative bacterium *Escherichia coli* CECT 434 and the Gram-positive bacteria *Staphylococcus aureus* CECT 976, *Enterococcus hirae* NCTC 13383 and *Listeria innocua* CECT 910 were used in this study. The bacteria were stored at −80 °C in Mueller-Hinton Broth (MHB; Merck, Darmstadt, Germany) supplemented with 30% glycerol (*v/v*) and subcultured in Mueller-Hinton agar (MHA, Merck) at 37 °C for 24 h. Prior to experiments, bacterial suspensions were grown overnight in MHB at 37 °C with shaking (120 rpm).

### 4.2. Photosensitizers and Light Source 

Stock solutions of rose bengal (RB; Sigma, St. Louis, MO, USA) and erythrosine (ERY; Sigma) were prepared in phosphate-buffered saline (PBS; pH 7.4) and stored at 4 °C in darkness until use. The solutions were spectrophotometrically standardized (Beckman Coulter DU *800 UV/Vis spectrophotometer, Brea, CA, USA). Prior to use, the photosensitizers (PS) solutions were allowed to warm to room temperature. The irradiation procedures were performed using a green light emitting diode (LED) apparatus, with a wavelength of 530 ± 40 nm and fluency rate of 10 mW/cm^2^. The LED beam profile was determined using a spectrofluorimeter (Varian Cary Eclipse, San Diego, CA, USA) and the intensity of the light was obtained with a Spectroradiometer USB2000+RAD (Ocean Optics, Winter Park, FL, USA). The equation applied by Gerola et al. [19] was used to calculate the LED beam array and the light doses of both dyes at the respective concentrations and irradiation time:

(1) $$D_{\mathrm{Abs}}=\frac{t}{A}.\int_{\lambda 1}^{\lambda 2}P_{\mathrm{Abs}}d\lambda$$

in which, *A* represents the irradiated area, *t* is the irradiation time, and P_Abs_ was obtained from the following equation:

(2) $$P_{\mathrm{Abs}}=X_{\mathrm{Abs}}P_{\mathrm{LED}\;\mathrm{Emitted}}$$

where X_Abs_ is the light fraction absorbed by the PS, P_Abs_ is the potency absorbed by the PS, and P_LED Emitted_ denotes the LED potency.

### 4.3. Photosensitization of Planktonic Cells

The experiments were performed as described by Penha et al. [15] with slight modifications. An overnight culture of each bacterium was centrifuged at 3772× *g* for 10 min and washed three times with 0.85% saline solution. The cell pellets were resuspended in PBS and the OD_620 nm_ of bacterial suspensions was adjusted to 0.1 ± 0.02. Afterward, 450 μL of these suspensions were dropped into a 24-well microtiter plate (Orange Scientific, Braine-l’Alleud, Belgium) and 50 μL of RB or ERY at several concentrations (from 1 to 50,000 nmol/L) were added. The microtiter plate was incubated at room temperature in the dark for 10 min and then irradiated for 10 min. Bacterial culturability was determined after 10-fold serial dilution and plating onto Plate Count Agar (PCA; Oxoid, Basingstoke, UK) plates. The number of colony forming units per milliliter (CFU/mL) was determined after incubation at 37 °C for 24 h. The controls included the incubation of bacteria in the presence of PS but without light irradiation (L−PS+) or in the absence of PSs but after light irradiation (L+PS−). The minimum bactericidal concentration (MBC) was considered the lowest concentration of the PS where no culturable cells could be recovered.

#### 4.3.1. Sessile Drop Contact Angle Measurements 

The physicochemical properties of the photoinactivated-bacterial surface as well as their respective controls (dark and irradiated) were determined by the sessile drop contact angle measurement on bacterial lawns as described previously [61]. An OCA 15 Plus video-based optical contact angle measurement instrument (DATAPHYSICS Instruments, Filderstadt, Germany) was used for contact angle determination. The measurements were performed using two polar solvents, water and formamide, and a nonpolar solvent, α-bromonaphthalene [62]. The liquids surface tension parameters were obtained from the literature [63]. The surface hydrophobicity was determined from contact angles data as proposed by van Oss [64,65,66] where the degree of hydrophobicity of a given surface is expressed as the free energy of interactions between two entities of that material immersed in water (ΔG_iwi_ mJ m^−2^). The ΔG_iwi_ can be calculated from the surface tension parameters of the interacting entities according to the following equation:

(3) $$\Delta G_{\mathrm{iwi}}=-2{\left(\sqrt{\gamma_{i}^{\mathrm{LW}}}-\sqrt{\gamma_{w}^{\mathrm{LW}}}\right)}^{2}+4\left(\sqrt{\gamma_{i}^{+}\gamma_{w}^{-}}+\sqrt{\gamma_{i}^{-}\gamma_{w}^{+}}-\sqrt{\gamma_{i}^{+}\gamma_{i}^{-}}-\sqrt{\gamma_{w}^{+}\gamma_{w}^{-}}\right)$$

in which γ^LW^ denotes for the Lifshitz–van der Waals component of the surface free energy and γ^−^ and γ^+^ are the donor and acceptor parameters, respectively, of the Lewis acid-base γ^AB^ parameters, where $\gamma^{\mathrm{AB}}=2\sqrt{\gamma^{+}\gamma^{-}}$. The surface tension parameters data can be obtained by the simultaneous resolution of the equation:

(4) $$\left(1+\cos\theta \right)\gamma_{i}^{\mathrm{Tot}}=2\left(\sqrt{\gamma_{s}^{\mathrm{LW}}\gamma_{i}^{\mathrm{LW}}}+\sqrt{\gamma_{s}^{+}\gamma_{i}^{-}}+\sqrt{\gamma_{s}^{-}\gamma_{i}^{+}}\right)$$

where *θ* represents the contact angle and $\gamma^{\mathrm{Tot}}={\;\gamma }^{\mathrm{LW}}+\gamma^{\mathrm{AB}}$.

#### 4.3.2. Potassium Leakeage Determination

The potassium (K^+^) leakage analysis was performed using flame emission and atomic absorption spectroscopy [33]. The planktonic bacterial cells were photoinactivated using the MBC. Then, 30 mL of cell suspension was filtered through a 0.45 μm pore size sterile cellulose nitrate membrane filter (Advantec, Toyo Roshi Kaisha, Ltd., Tokyo, Japan) and 0.25 g of NaCl was added to avoid another loads interference. The samples were analyzed in a GBC AAS 932 plus device using GBC Avante 1.33 software (GBC Scientific Equipment, Hampshire, IL, USA). L−PS+ and L+PS− exposed cells were used as controls.

### 4.4. Photosensitization of Biofilm Cells

Biofilms were formed according to Stepanović et al. [67] with slight modifications. An overnight culture of each bacterium was diluted in fresh MHB (~10^7^ CFU/mL) and 250 μL of the suspension was transferred to 24-well polystyrene microtiter plates to form biofilms at 37 °C and under stirring (150 rpm) for 24 h. This period of incubation was chosen according to previous studies demonstrating the biofilm formation of food-related bacterial strains after 24 h [68,69]. Afterwards, the medium was removed, the wells gently washed with 0.85% saline solution and 500 μL of RB (from 0.01 to 10 μmol/L) or ERY (from 0.01 to 50 μmol/L) solutions were added to the wells. Plates were irradiated with green LED light source for 30 min after dark incubation for 30 min. After photosensitization, the wells were washed once and the plates were submitted to ultrasonic bath (35 kHz for 3 min). The resulting cell suspensions were enumerated by colony counts and their membrane integrity assessed by live/dead staining.

#### 4.4.1. Enumeration of Culturable Cells 

After the PDI treatment, the cell suspensions were serially diluted in 0.85% saline solution and 10 µL droplets of each dilution were plated on PCA plates in order to determine the number of culturable cells (CFU/cm^2^). After drying, the plates were incubated at 37 °C for 24 h and the CFU were counted. PS-treated biofilms not exposed to LED irradiation and PS-free biofilms exposed to light were used as controls.

#### 4.4.2. Assessment of Membrane Integrity of Biofilm Cells 

The bacterial membrane integrity was assessed by fluorescence microscopy using the Live/Dead (LD) *Bac*Light^®^ kit. This methodology uses a mixture of two dyes (SYTO-9 and Propidium iodide [PI]) that allows the assessment of the membrane integrity by selective stain exclusion [70]. For this purpose, 300 μL of biofilm suspensions were filtered through a Nucleopore^®^ (Whatman International Ltd., Maidstone, UK) black polycarbonate membrane (pore size 0.22 μm) and stained with LD dyes, according to the manufacturer’s instructions. Afterwards, the samples were observed in a DMLB2 microscope (LEICA Microsystems Ltd., Weltzlar, Germany) equipped with a HBO/100W/3 mercury lamp. Images were captured using a CCD camera and IM50 software (LEICA). The percentage of green (stained with SYTO-9) and red cells (stained with both SYTO-9 and PI) was quantified from 20 microscopy fields. Controls included dark and irradiation treatments.

### 4.5. Statistics

Statistical analysis was performed using GraphPad Prism 7.0 (GraphPad Software, San Diego, CA, USA). The assays were carried out in duplicate and repeated three times. One-way analysis of variance (ANOVA) followed by post hoc Tukey’s test was applied; when the assumptions for the parametric test were not respected, Kruskal-Wallis non-parametric test followed by Dunn’ multiple comparison tests was performed. *p*-values lower than 0.05 were considered statistically significant.

## 5. Conclusions

It was demonstrated that RB and ERY are able to inactivate *S. aureus*, *L. innocua*, *E. hirae* and *E. coli* in both planktonic and sessile states upon illumination with green LED light. *L. innocua* was the most susceptible to aPDI while *E. coli* was the most tolerant. The photokilling process changed the surfaces properties of the bacterial cells and induced K^+^ leakage. Higher PS concentration and longer irradiation exposure were needed to control sessile cells, which can be mainly associated with the limited penetration of the PS and light through the biofilm. Generally, RB presented better antimicrobial/antibiofilm effects compared to ERY, which may be due to the high singlet oxygen quantum yield of that PS. It is worth to mention that the light doses calculations took into account the photons absorbed by the PS and that those values changed according to the type and concentration of the PS and to the time of light delivered. Worryingly, several studies do not consider such parameters, making difficult the direct comparison between photodynamic studies. The overall results further support the conclusion that RB and ERY present potent antimicrobial and antibiofilm effect and may be potentially applied in the food sector for microbial control.

## Figures and Tables

**Table 1:** Light dose values (J/cm^2^) obtained with different PS concentrations and light exposure times.

Planktonic Cells ^a^			Biofilms ^b^		
PS Concentration (nmol/L)	Light Dose		PS Concentration (μmol/L)	Light Dose	
	ERY	RB		ERY	RB
10.0	0.22	0.17	0.010	0.65	0.53
25.0	0.55	0.43	0.025	1.53	1.31
50.0	1.21	0.87	0.050	3.34	2.62
75.0	1.67	1.31	0.075	4.99	3.93
100.0	2.54	1.74	0.100	7.61	5.24
250.0	5.47	4.33	0.250	16.41	12.99
500.0	10.73	8.55	0.500	32.30	25.65
750.0	15.77	12.61	0.750	47.31	38.00
1000.0	20.52	16.54	1.000	61.56	49.59
5000.0	77.14	57.79	2.500	138.13	113.43
10,000.0	113.81	102.98	3.000	-	132.24
15,000.0	132.24	125.40	5.000	231.42	197.41
20,000.0	141.17	139.46	7.000	-	249.28
25,000.0	148.01	147.06	7.500	295.83	260.49
50,000.0	162.07	166.44	9.000	-	291.08
-	-	-	10.00	338.58	308.75
-	-	-	20.00	423.51	-
-	-	-	25.00	444.03	-
-	-	-	30.00	462.27	-
-	-	-	35.00	471.96	-
-	-	-	40.00	478.80	-
-	-	-	50.00	486.40	-

^a.^ 10 min of light exposure; ^b.^ 30 min of light exposure; - not evaluated.

**Table 2:** Hydrophobicity (∆G_iWi_), apolar (γ^LW^) and polar (γ^AB^) components of the surface tension of untreated and aPDI planktonic bacteria.

Bacteria	Treatment	Hydrophobicity (mJ/m^2^)	Surface Tension Parameters (mJ/m^2^)			
		ΔG_iWi_	γ^LW^	γ^+^	γ^−^	γ^AB^
*E. coli*	Control	24.48 ± 1.21 ^a^	25.41 ± 0.16 ^a^	5.06 ± 0.35 ^a^	52.68 ± 0.72 ^a^	32.63 ± 0.90 ^a^
	Light	23.30 ± 0.62 ^a^	25.23 ± 1.63 ^a^	5.19 ± 0.83 ^a^	51.49 ± 0.91 ^a^	32.65 ± 2.90 ^a^
	ERY ^(L−P+)^	20.06 ± 2.58 ^b^	21.83 ± 2.06 ^a^	7.12 ± 1.23 ^a^	51.17 ± 0.95 ^a^	38.09 ± 2.95 ^a^
	ERY ^(L+P+)^	27.43 ± 0.69 ^a^	30.98 ± 1.09 ^b^	3.03 ± 0.37 ^a^	52.46 ± 0.12 ^a^	25.19 ± 1.51 ^b^
	RB ^(L−P+)^	25.01 ± 1.73 ^a^	23.24 ± 0.35 ^a^	5.54 ± 0.34 ^a^	54.34 ± 1.47 ^a^	34.68 ± 0.60 ^a^
	RB ^(L+P+)^	28.55 ± 1.77 ^a^	30.28 ± 1.14 ^b^	2.87 ± 0.21 ^b^	53.03 ± 2.14 ^a^	24.66 ± 1.38 ^b^
*S. aureus*	Control	29.57 ± 2.32 ^a^	31.83 ± 0.40 ^a^	2.03 ± 0.44 ^a^	52.05 ± 1.11 ^a^	20.46 ± 2.02 ^a^
	Light	36.28 ± 1.61 ^b^	32.64 ± 0.23 ^a^	1.32 ± 0.05 ^a^	56.48 ± 1.69 ^a^	17.26 ± 0.60 ^a^
	ERY ^(L−P+)^	30.65 ± 2.18 ^a^	31.51 ± 0.37 ^a^	2.35 ± 0.33 ^a^	54.06 ± 1.38 ^a^	22.50 ± 1.28 ^a^
	ERY ^(L+P+)^	21.76 ± 0.86 ^b^	27.95 ± 4.58 ^a^	4.01 ± 0.91 ^a^	47.78 ± 1.71 ^b^	27.64 ± 3.66 ^b^
	RB ^(L−P+)^	32.00 ± 2.14 ^a^	31.94 ± 0.87 ^a^	2.13 ± 0.46 ^a^	54.90 ± 0.98 ^a^	21.58 ± 2.16 ^a^
	RB ^(L+P+)^	22.44 ± 0.47 ^b^	28.40 ± 0.62 ^a^	3.64 ± 0.62 ^a^	47.66 ± 0.64 ^b^	26.29 ± 2.42 ^b^
*E. hirae*	Control	32.50 ± 1.45 ^a^	33.47 ± 0.95 ^a^	1.53 ± 0.10 ^a^	53.95 ± 1.41 ^a^	18.18 ± 0.33 ^a^
	Light	34.69 ± 0.14 ^a^	34.23 ± 0.19 ^a^	1.35 ± 0.09 ^a^	55.66 ± 0.40 ^a^	17.36 ± 0.62 ^a^
	ERY ^(L−P+)^	27.85 ± 0.96 ^a^	26.76 ± 0.63 ^a^	3.79 ± 0.19 ^a^	53.82 ± 1.52 ^a^	28.56 ± 1.13 ^a^
	ERY ^(L+P+)^	30.04 ± 0.04 ^a^	32.07 ± 2.10 ^a^	2.18 ± 0.41 ^a^	53.14 ± 0.49 ^a^	21.47 ± 2.15 ^a^
	RB ^(L−P+)^	27.41 ± 0.67 ^a^	30.02 ± 0.31 ^a^	2.96 ± 0.10 ^a^	51.93 ± 0.89 ^a^	24.78 ± 0.63 ^a^
	RB ^(L+P+)^	34.38 ± 3.05 ^a^	33.39 ± 0.71 ^a^	1.56 ± 0.32 ^a^	55.81 ± 1.50 ^a^	18.60 ± 1.68 ^a^
*L. innocua*	Control	29.97 ± 4.83 ^a^	27.80 ± 0.82 ^a^	2.96 ± 0.78 ^a^	53.98 ± 3.22 ^a^	29.98 ± 2.60 ^a^
	Light	32.79 ± 3.53 ^a^	28.98 ± 3.13 ^a^	2.30 ± 0.94 ^a^	55.28 ± 1.43 ^a^	32.80 ± 4.38 ^a^
	ERY ^(L−P+)^	31.54 ± 3.38 ^a^	27.67 ± 1.86 ^a^	2.88 ± 0.99 ^a^	55.43 ± 1.09 ^a^	31.55 ± 4.12 ^a^
	ERY ^(L+P+)^	26.32 ± 3.77 ^a^	32.05 ± 1.94 ^a^	2.62 ± 0.09 ^a^	50.60 ± 2.97 ^a^	26.32 ± 0.30 ^a^
	RB ^(L−P+)^	31.66 ± 3.08 ^a^	29.95 ± 0.42 ^a^	2.41 ± 0.53 ^a^	54.76 ± 1.69 ^a^	31.66 ± 2.17 ^a^
	RB ^(L+P+)^	27.40 ± 1.91 ^a^	30.44 ± 3.23 ^a^	2.86 ± 0.90 ^a^	51.77 ± 0.66 ^a^	27.40 ± 3.69 ^a^

Different letters (a or b) in the same column indicate statistical difference between means (*p* < 0.05). ^(L-P+)^ Treatment in the presence of the photosensitizer without light exposure; ^(L+P+)^ Treatment using the combination of photosensitizer and irradiation. The control group represents the cells incubated only with PBS. Values are the mean ± SD of three independent experiments.

**Table 3:** Effect of photoinactivation mediated by RB and ERY on K^+^ leakage (mg/L) of the bacteria tested.

	*E. coli*	*S. aureus*	*E. hirae*	*L. innocua*
Control	0.00 ± 0.00	0.00 ± 0.00	0.00 ± 0.00	0.00 ± 0.00
Light	0.00 ± 0.00	0.00 ± 0.00	0.00 ± 0.00	0.00 ± 0.00
ERY ^(L−P+)^	0.00 ± 0.00	0.00 ± 0.00	0.00 ± 0.00	0.00 ± 0.00
ERY ^L+P+)^	0.64 ± 0.06 *	1.23 ± 0.03 *	1.09 ± 0.09 *	0.86 ± 0.18 *
RB ^(L−P+)^	0.00 ± 0.00	0.00 ± 0.00	0.00 ± 0.00	0.00 ± 0.00
RB ^L+P+)^	0.20 ± 0.03 *	1.22 ± 0.16 *	0.52 ± 0.09 *	0.53 ± 0.14 *

^(L−P+)^ Treatment in the presence of the PS without light exposure; ^(L+P+)^ Treatment using the combination of PS and irradiation. The control group represents the cells incubated only with PBS. Values are the means ± SD of three independent experiments. * *p* < 0.05 compared to the control.

**Table 4:** Percentage of biofilm cells stained with propidium iodide.

	*E. coli*	*S. aureus*	*E. hirae*	*L. innocua*
Control	6.72 ± 3.14	1.65 ± 2.85	2.10 ±3.27	9.47 ± 4.56
ERY ^a^	7.37 ± 4.91	5.46 ± 7.06	0.41 ± 1.29	17.2 ± 12.3
ERY ^b^	98.2 ± 3.68 *	100 ± 0.00 *	100 ± 0.00 *	100 ± 0.00 *
RB ^a^	1.46 ± 2.94 *	6.06 ± 8.38	2.20 ± 2.67	2.98 ± 4.44
RB ^c^	99.5 ± 1.51 *	100 ± 0.00 *	100 ± 0.00 *	99.1 ± 1.77 *

^a^ Minimum concentration of PS evaluated on CFU assay (0.10, 0.01, 0.025 and 0.01 μmol/L for *E. coli*, *S. aureus*, *E. hirae* and *L. innocua*, respectively); ^b^ ERY at minimum biofilm eradication concentration observed by CFU counts (50, 7.5, 0.75, 1.0 μmol/L for *E. coli*, *S. aureus*, *E. hirae* and *L. innocua*, respectively); ^c^ RB at minimum biofilm eradication concentration observed by CFU counts (9.0, 1.0, 0.75, 0.20 μmol/L for *E. coli*, *S. aureus*, *E. hirae* and *L. innocua*, respectively). The control group represents the cells incubated only with PBS. Values are the mean ± SD of three independent experiments. * *p* < 0.05 compared to the control.

## Supplementary Materials

The supplementary materials are available online.
