# Supplementary material for: Antimicrobial Photodynamic Inactivation Mediated by Rose Bengal and Erythrosine Is Effective in the Control of Food-Related Bacteria in Planktonic and Biofilm States
Source: Molecules. 2018 Sep 7;23(9):2288. doi: 10.3390/molecules23092288 (PMC6225188; doi:10.3390/molecules23092288)
Supplement: Supplementary file 1 [file molecules-23-02288-s001.pdf]

Supplementary material

Antimicrobial photodynamic inactivation mediated by rose bengal and erythrosine is effective in the control of food-related bacteria in planktonic and biofilm states

Alex Fiori Silva [1,2], Anabela Borges[2], Camila Fabiano Freitas[3], Noboru Hioka[3], Jane Martha Graton Mikcha[1], Manuel Simões [2,*]

1 PostGraduate Program of Health Sciences, State University of Maringá, Maringá, Paraná, Brazil, Av. Colombo, 5790, 87020-900 Maringá, Brazil; alex_skiba@hotmail.com (A.F.S); jmgmikcha@uem.br (J.M.G.M)

2 LEPABE, Department of Chemical Engineering, Faculty of Engineering, University of Porto, Rua Dr. Roberto Frias, s/n, 4200-465 Porto, Portugal; apborges@utad.pt (A.B); mvs@fe.up.pt (M.S)

3 [c]Department of Chemistry, State University of Maringa, Maringá, Paraná, Brazil, Av. Colombo, 5790, 87020-900 Maringá, Brazil; camila.freitas1989@hotmail.com (C.F.F); nhioka@uem.br (N.H)

*Author to whom correspondence should be addressed.

Light doses calculations

The absolute irradiance of the LEDs was evaluated in a Spectroradiometer obtained by Ocean Optics model USB2000 +. The power absorbed (PAbs) and number of absorbed photons (NAbs) were performed following the methodology described previously [19,20].

The number of photons emitted (NEm) by a monochromatic light source (LASER), was calculated by Equation S1, corresponding to a given energy inherent to the photon frequency:

$$N_{em} = \frac{E}{h\upsilon} \qquad \text{(Equation S1)}$$

where E is the energy (in J), h is the Planck constant (6.626×10^{-34}, in J s) and υ is the frequency (in s^{-1}). Equation S1 can be rearranged to provide Equation S2, which includes Avogadro's number (Na: 6.022×10^{23}/ mol) to represent the equation in number of photon moles emitted (equivalent to Einsteins):

$$N_{em} = \frac{E}{h\upsilon} = \frac{P_{em}t}{h\left(\frac{c}{\lambda}\right)Na} = \frac{\lambda P_{em}t}{hcNa} \qquad \text{(Equation S2)}$$

Equation S2 gives the number of photon moles (Einsteins) emitted by a monochromatic light source. When using polychromatic light sources (such as LEDs), light irradiation must be considered throughout the spectral region. Thus, the emitted power (PEm) can be obtained by Equation S3:

$$I \approx P_{em} = \int_{\lambda_i}^{\lambda_f} I_0(\lambda')d\lambda \qquad \text{(Equation S3)}$$

Where λi and λf are the initial and final wavelengths of the LED irradiation spectrum, respectively. Substituting the value of Pem in Equation S3 it is obtained:

$$N_{em} = \frac{t}{hcNa}\int_{\lambda_i}^{\lambda_f} I_0(\lambda')d\lambda' \qquad \text{(Equation S4)}$$

The fraction of light absorbed by a given sample can be defined as:

$$X_{abs} = 1 - 10^{-Abs} \qquad \text{(Equation S5)}$$

Equation S5 can be used only for a specific λ, it means, monochromatic irradiation sources. For polychromatic sources, such as LEDs, it is necessary to consider the value of X_{Abs} over the entire spectrum of electronic absorption of PS. For that Equation S6 considers the light fraction throughout the spectral region.

$$\int X_{Abs}(\lambda')d\lambda' = \int 1 - 10^{-Abs(\lambda')}d\lambda' \qquad \text{(Equation S6)}$$

The absorbed power (P_{Abs}) results from the product between the total power emitted and the absorbed light fraction (Equation S7).

$$P_{Abs} = P_{em}X_{Abs} \qquad \text{(Equation S7)}$$

For a polychromatic light source P_{Abs} is defined by Equation S8.

$$P_{Abs} = \int_{\lambda i}^{\lambda f} I_0(\lambda')X_{Abs}(\lambda')d\lambda' \qquad \text{(Equation S8)}$$

Thus, these terms can be inserted in Equation S4, number of photons emitted (NEm) to obtain the equation of the number of absorbed photons (NAbs). Therefore, to determine the number of photons absorbed by a PS employing a monochromatic irradiation source, the term Pem is replaced by the term P_{Abs} in Equation S4, obtaining Equation S9 (in Einsteins):

$$N_{Abs} = \frac{P_{em}X_{Abs}\lambda t}{hcNa} = \frac{P_{Abs}\lambda t}{hcNa} \qquad \text{(Equation S9)}$$

For polychromatic irradiation sources, it is necessary to consider the power absorbed by the entire LED/PS overlap spectral region:

$$N_{Abs} = \frac{t}{hcNa}\int_{\lambda i}^{\lambda f} I_0(\lambda')X_{Abs}(\lambda')\lambda(\lambda')d\lambda' \quad \text{(Equation S10)}$$

Finally, it is possible to obtain the actual light dose (J/cm^2) absorbed by the PS from Equation S11:

$$D_{Abs} = \frac{t}{A}\int_{\lambda_1}^{\lambda_2} P_{Abs} d\lambda \quad \text{(Equation S11)}$$

in which A is the irradiated area, t is the illumination time, P_{Abs} is the power absorbed by the PS.

Table S1. Number of absorbed photons, NAbs (10^{-9} mol), obtained with different PS concentrations and light exposure times.

Planktonic cells[a]			Biofilms[b]		
	NAbs (10^{-9} mol)			NAbs (10^{-9} mol)	
PS concentration (nmol/L)	ERY	RB	PS concentration (μmol/L)	ERY	RB
10.0	1.0	0.8	0.010	3.0	2.3
25.0	2.3	1.9	0.025	6.8	5.8
50.0	4.9	3.9	0.050	14.8	11.7
75.0	7.4	5.8	0.075	2.21	17.4
100.0	9.8	7.7	0.100	29.4	23.2
250.0	24.2	19.2	0.250	72.7	57.5
500.0	47.5	37.8	0.500	142.5	113.3
750.0	69.8	55.8	0.750	209.7	167.3
1000.0	91.3	73.2	1.000	274.0	219.6
5000.0	341.3	290.9	2.500	611.1	501.9
10000.0	502.9	455.3	3.000	-	585.1
15000.0	573.3	554.1	5.000	1024.0	872.9
20000.0	633.6	616.7	7.000	-	110.4
25000.0	662.3	650.9	7.500	1308.2	1152.3
50000.0	712.9	738.2	9.000	-	1287.2
-	-	-	10.00	1508.8	1365.9
-	-	-	20.00	1900.7	1850.2
-	-	-	25.00	1986.8	1952.8
-	-	-	30.00	2042.0	2057.1
-	-	-	35.00	2082.9	2116.1
-	-	-	40.00	2111.4	2158.5
-	-	-	50.00	2138.8	2214.5

[a]10 min of light exposure; [b]30 min of light exposure; - not evaluated.

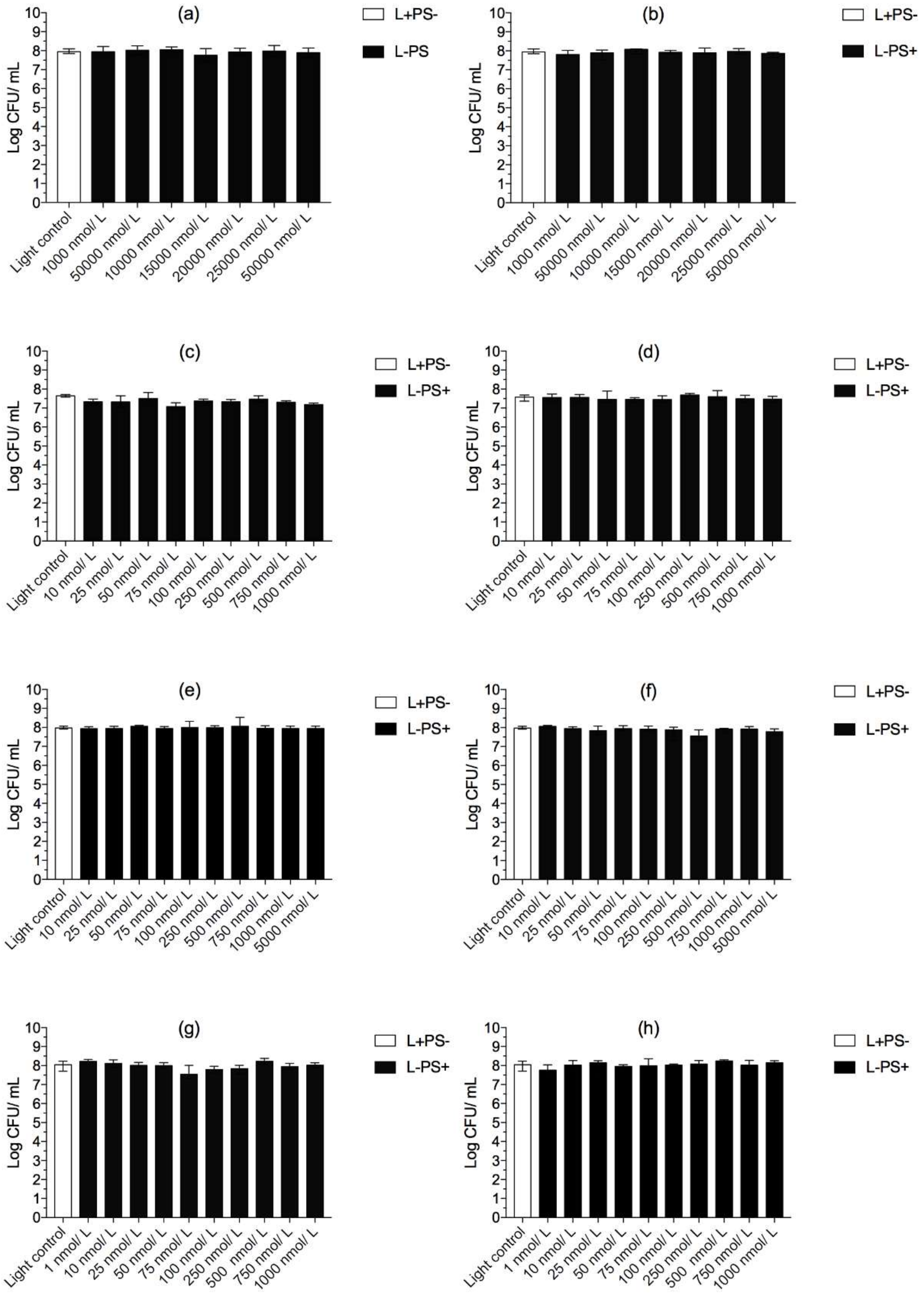

Figure S1. Survival of (a,b) *E. coli*, (c,d) *S. aureus*, (e,f) *E. hirae* and (g,h) *L. innocua* planktonic cells exposed only to the light source during 20 min or only to RB (left) and ERY (right). Values are shown as medians, including 25 and 75% quantiles of at least three independent experiments.

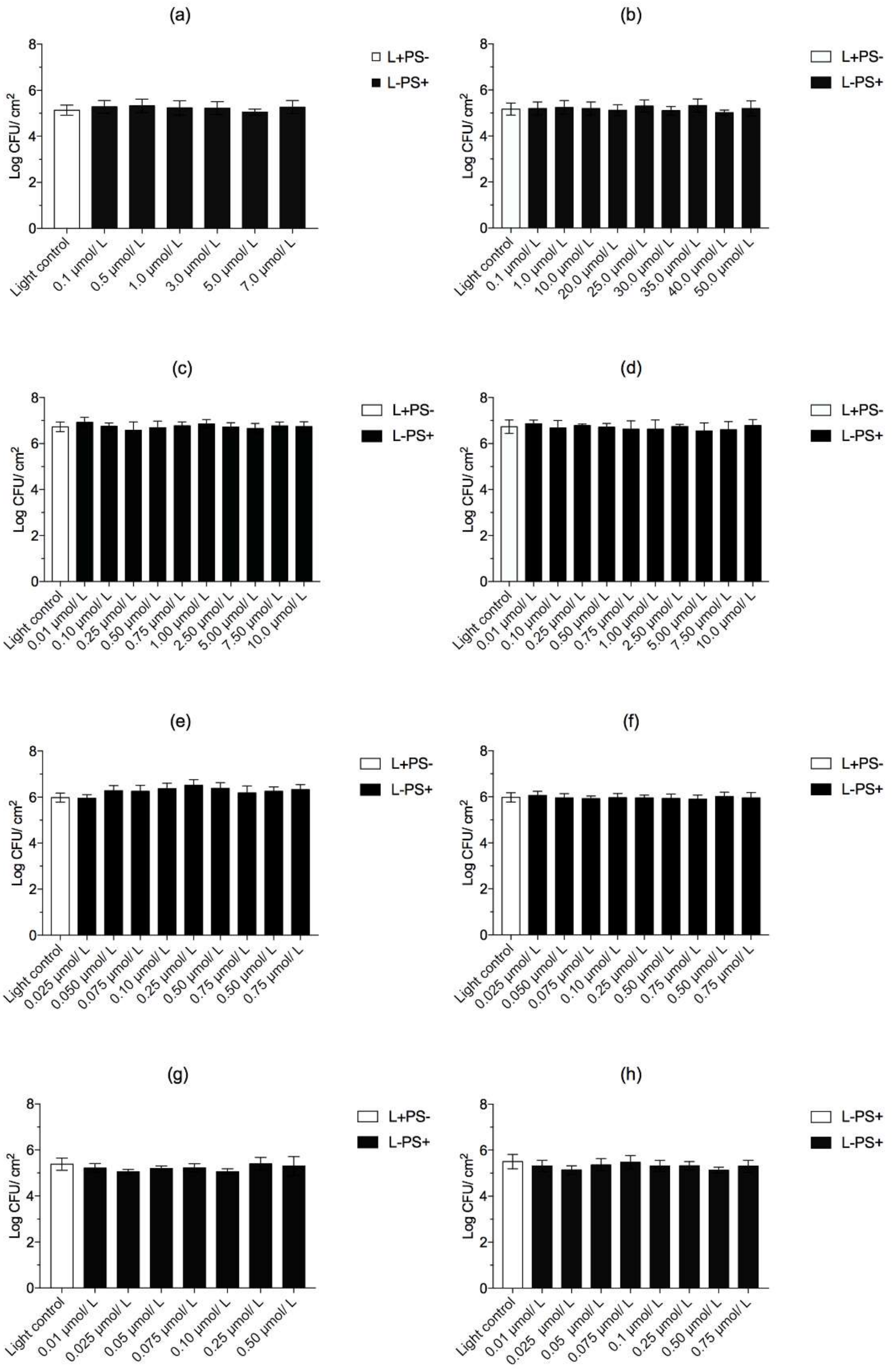

Figure S2. Survival of (a,b) *E. coli,* (c,d) *S. aureus,* (e,f) *E. hirae* and (g,h) *L. innocua* biofilms cells exposed only to the light source during 60 min or only to RB (left) and ERY (right) for 60 min. Values are shown as medians, including 25 and 75% quantiles of at least three independent experiments.
